# Forsythoside A alleviates experimental autoimmune encephalomyelitis by targeting Tnfaip2

**DOI:** 10.1186/s13020-026-01476-z

**Published:** 2026-07-29

**Authors:** Ping Tang, Guibao Luo, Huihui Xue, Chunsheng Yang

**Affiliations:** 1 Department of Neurology, Tianjin Neurological Institute, Tianjin Medical University General Hospital, Tianjin, China; 2 Department of Neurology and Neurological Rehabilitation, Shanghai Disabled Persons’ Federation Key Laboratory of Intelligent Rehabilitation Assistive Devices and Technologies, Yangzhi Rehabilitation Hospital (Shanghai Sunshine Rehabilitation Center), School of Medicine, Tongji University, Shanghai, China

**Keywords:** Forsythoside A, Experimental autoimmune encephalomyelitis, Microglial polarization, Tnfaip2, NF-κB pathway

## Abstract

**Background:**

Multiple sclerosis (MS) is a debilitating neuroinflammatory disease. Experimental autoimmune encephalomyelitis (EAE) is a commonly used rodent model for MS. Forsythoside A (FA), a natural compound derived from *Forsythia suspensa*, exhibits anti-inflammatory and neuroprotective properties; however, its efficacy in EAE and its underlying mechanism remain unclear.

**Purpose:**

To explore the therapeutic effect of FA on EAE and its mechanism.

**Methods:**

EAE was induced in C57BL/6 mice treated with FA (20/60 mg/kg) from day 7. Clinical scores, histopathology (H&E, LFB, immunofluorescence), flow cytometry, and spinal cord RNA sequencing were analyzed. Network pharmacology, molecular docking, molecular dynamics (MD) simulations, and surface plasmon resonance (SPR) were employed to identify and validate the target of FA. In vitro studies using BV2 microglia with Tnfaip2 knockdown/overexpression confirmed the mechanism via qRT-PCR and Western blot.

**Results:**

FA treatment dose-dependently alleviated clinical severity. While the 20 mg/kg dose showed a trend toward improvement without statistical significance, the 60 mg/kg dose significantly reduced CNS inflammation and demyelination. FA decreased peripheral Th1 cells in EAE mice. It suppressed microglial activation and pro-inflammatory markers (IL-1β, TNF-α, iNOS) expression. Transcriptomics and network analysis pinpointed TNF-α-induced protein 2 (Tnfaip2) in the TNF pathway. FA directly bound Tnfaip2 (K_D_ = 28 µM), validated by docking, MD simulations, and SPR. In vitro, FA inhibited LPS-induced pro-inflammatory polarization and the Tnfaip2/NF-κB pathway in BV2 cells. Tnfaip2 overexpression abolished the anti-inflammatory effects of FA.

**Conclusion:**

FA ameliorates EAE by suppressing pro-inflammatory microglial polarization via directly targeting Tnfaip2 and inhibiting the NF-κB pathway. This identifies the Tnfaip2/NF-κB axis as a potential therapeutic target for MS.

**Supplementary Information:**

The online version contains supplementary material available at 10.1186/s13020-026-01476-z.

## Introduction

Multiple sclerosis (MS) is an inflammatory demyelinating disease of the central nervous system (CNS) and represents one of the leading causes of neurological disability in young adults worldwide [1]. The characteristic neuropathological hallmarks of MS include inflammation, demyelination, axonal loss or injury, and gliosis [2]. Although the precise mechanisms underlying the pathogenesis of MS remain incompletely understood, it is widely regarded as an autoimmune disorder mediated primarily by CD4^+^ T cells [3]. Upon crossing the blood–brain barrier, activated Th1 and Th17 cells encounter antigen-presenting cells within the CNS. When these autoreactive, peripherally primed T cells recognize autoantigenic peptides in the brain parenchyma, they become reactivated, triggering an inflammatory cascade. This process leads to the release of cytokines and chemokines, which recruit additional inflammatory cells. Persistent activation of microglia and macrophages subsequently contributes to myelin damage [4–6]. As many components of this cascade have been identified, tested, and validated in the experimental autoimmune encephalomyelitis (EAE) model, EAE serves as a highly relevant and widely used experimental model for studying the pathogenesis of MS [7–9].

Forsythoside A (FA, C_29_H_36_O_15_) is one of the primary active constituents isolated from the dried fruits of *Forsythia suspensa*. It has been reported to possess various pharmacological activities, including anti-inflammatory, antioxidant, and antiviral effects [10–12]. Owing to its ability to cross the blood–brain barrier via both passive diffusion and active transport [13], FA has been investigated in therapeutic contexts for animal models of Alzheimer's disease [14], Parkinson's disease [15], and transient global cerebral ischemia [16]. However, whether FA confers any therapeutic benefit in EAE and its underlying mechanism of action remains unexplored. In this study, we investigated the protective properties of FA and its efficacy in suppressing pro-inflammatory microglial polarization in a rodent model of EAE.

## Results

### Therapeutic effects of FA on EAE

#### FA ameliorates spinal cord inflammation and demyelination in EAE

**Fig. 1:**
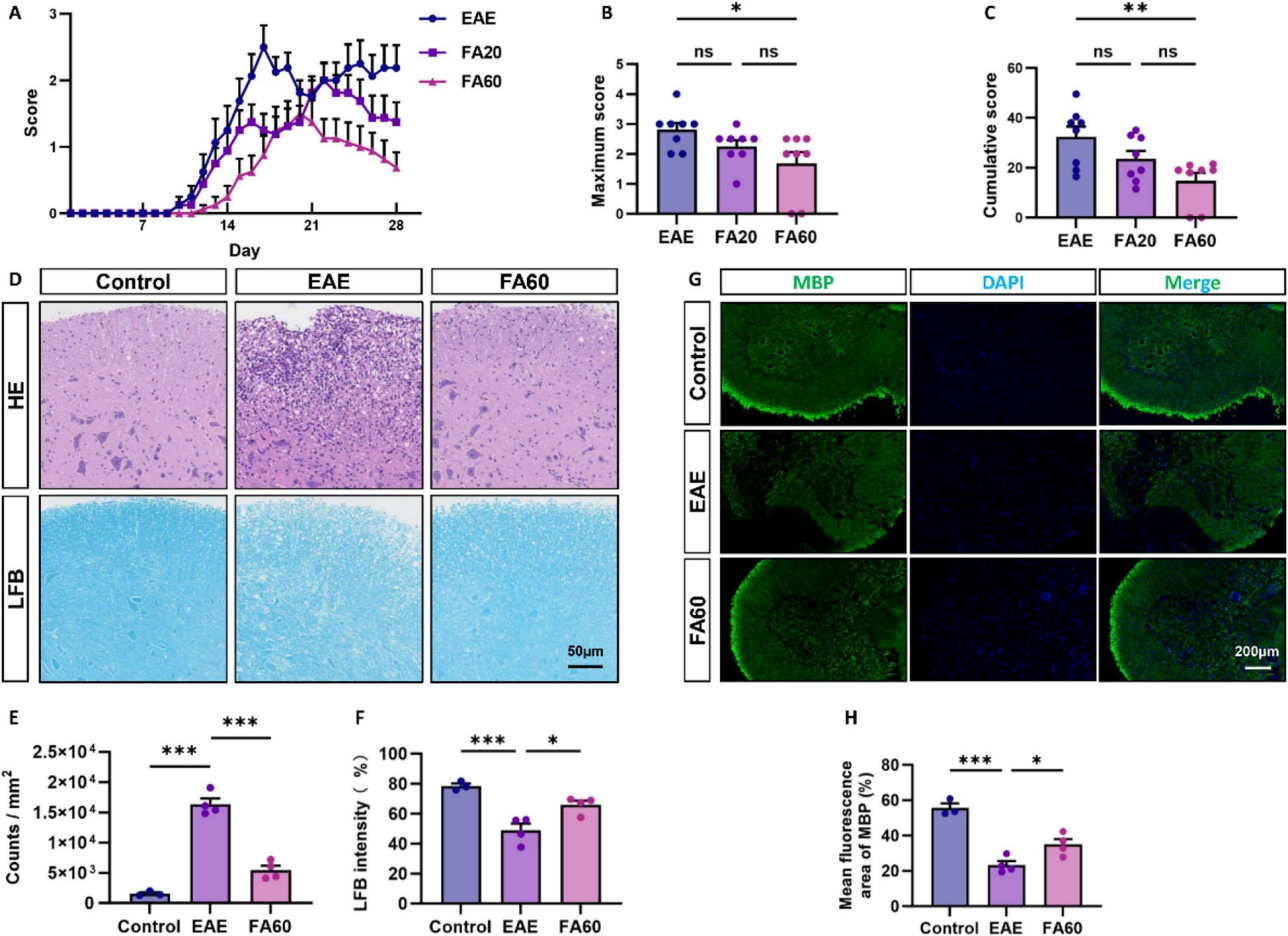
FA confers neuroprotective effects in EAE mice. **A** Neurological function scores of mice in each group (n = 8 per group). **B** Peak clinical scores of mice in each group. **C** Total clinical scores of mice in each group. **D** Representative images of H&E and LFB staining in the lumbar spinal cord (Control, n = 3; EAE, n = 4; FA60, n = 4). **E** Quantification of inflammatory cell infiltration in the lumbar spinal cord. **F** The proportion of demyelination in the lumbar spinal cord. **G** Representative immunofluorescence images of MBP in lumbar spinal cord cryosections (Control, n = 3; EAE, n = 4; FA60, n = 4). **H** Quantitative analysis of MBP percentage in the lumbar spinal cord. Data are presented as mean ± SEM. *p < 0.05, **p < 0.01, ***p < 0.001; *ns* not significant

Neurological function and behavioral scores were assessed daily in all experimental groups. Mice in the EAE model group began to exhibit clinical symptoms approximately 10 days post-immunization, characterized by gait ataxia, limb weakness or paralysis, loss of tail tone, and dull fur. Disease severity peaked around day 17 (Fig. 1A). In contrast, FA-treated mice showed a delayed disease onset and attenuated clinical severity compared to the EAE group. Statistical analysis of both cumulative and maximum clinical scores revealed that the low-dose FA group did not show significant differences compared to the EAE group (*p* = 0.2111 and *p* = 0.3558, respectively). However, significant improvements were observed in the high-dose FA group (*p* = 0.0058 and *p* = 0.0270, respectively). Consequently, only the high-dose FA group was selected for subsequent experiments (Fig. 1B, C).

Hematoxylin and eosin (H&E) staining of spinal cord sections (Fig. 1D, E) showed minimal inflammatory infiltration in controls, substantial infiltration in EAE mice, and a notable reduction in the FA-treated group. Furthermore, Luxol fast blue (LFB) staining (Fig. 1D, F) and myelin basic protein (MBP) immunofluorescence (Fig. 1G, H) confirmed extensive demyelination in the EAE model, which was significantly attenuated by FA administration.

#### FA-mediated attenuation of peripheral Th1 cells in EAE mice

**Fig. 2:**
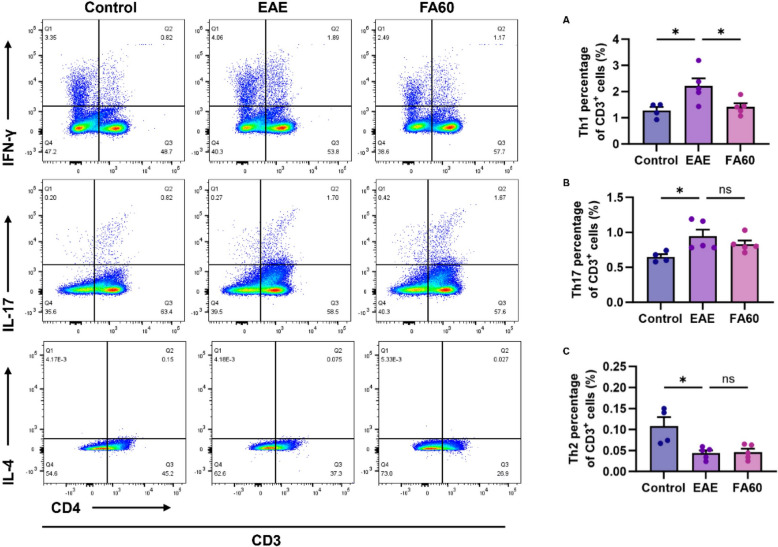
FA modulates the differentiation of peripheral Th cell subsets in EAE mice. **A** Proportion of Th1 cells in the spleen, determined by flow cytometry. **B** Proportion of Th17 cells in the spleen, determined by flow cytometry. **C** Proportion of Th2 cells in the spleen, determined by flow cytometry. Quantitative data are presented as mean ± SEM. *p < 0.05; *ns* not significant; Control, n = 4; EAE, n = 5; FA60, n = 5

Activated CD4 + T cells serve as primary drivers in the pathogenesis of EAE, with Th1 cells predominating in early inflammation and Th17 cells contributing to chronic disease progression. In contrast, Th2 cells play a regulatory role by suppressing Th1/Th17 responses and promoting tissue repair processes such as remyelination [17]. Flow cytometric analysis of splenocytes revealed that EAE mice exhibited increased differentiation of Th1 and Th17 cells, accompanied by a reduction in Th2 cell frequency. Treatment with FA significantly suppressed Th1 cell differentiation in the periphery (Fig. 2A), whereas it did not exert significant effects on the differentiation of Th17 or Th2 cells (Fig. 2B, C).

#### FA-mediated amelioration of neuroinflammation in the EAE mouse model

**Fig. 3:**
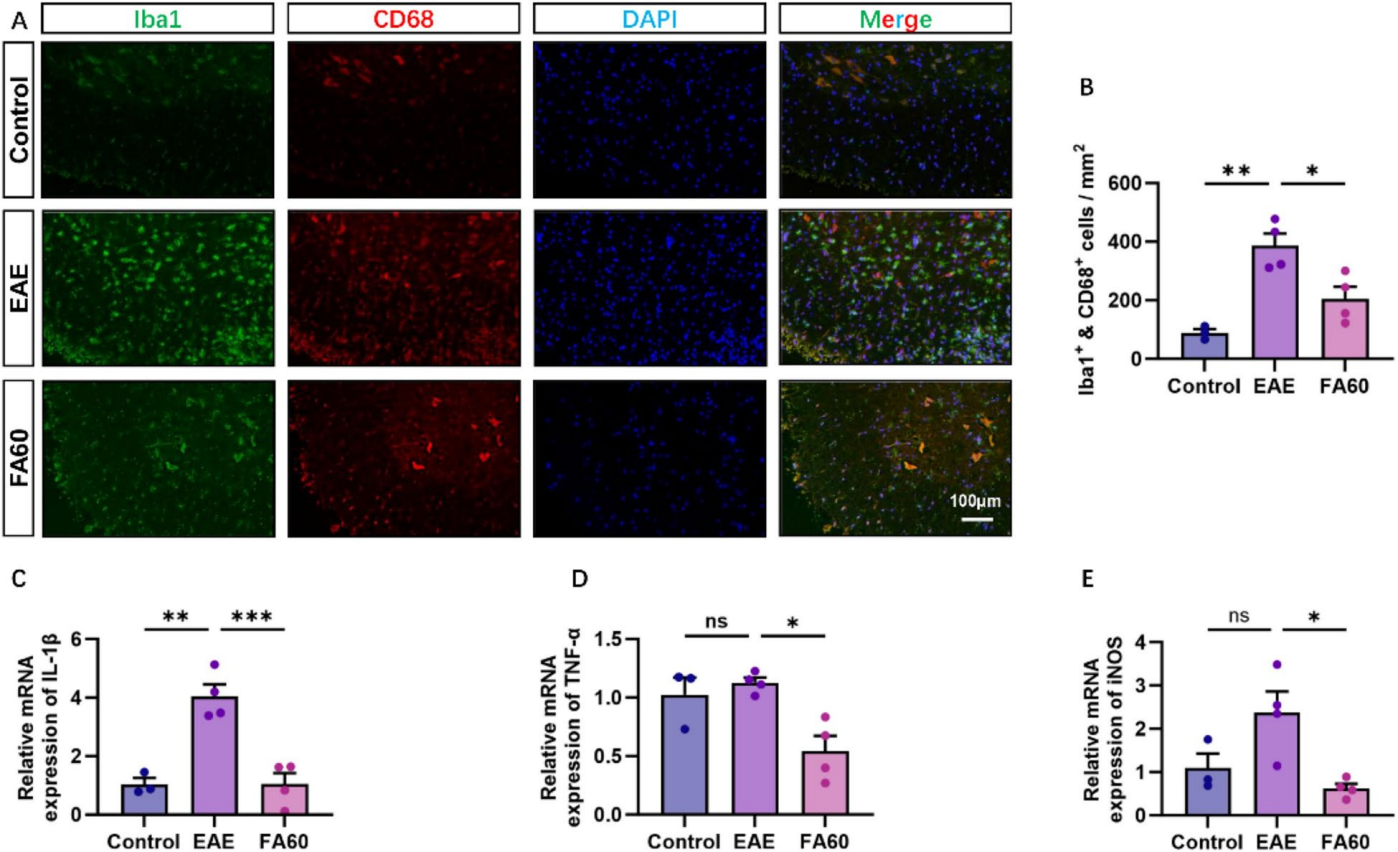
FA ameliorates microglial activation and neuroinflammation in the spinal cord of EAE mice. **A** Representative immunofluorescence images of microglia in the spinal cord. **B** Quantification of Iba1⁺ and CD68⁺ microglia in the spinal cord. **C** Expression level of IL-1β in the spinal cord at the peak disease stage. **D** Expression level of TNF-α in the spinal cord at the peak disease stage. **E** Expression level of iNOS in the spinal cord at the peak disease stage. Data are presented as mean ± SEM. *p < 0.05, **p < 0.01, ***p < 0.001; *ns* not significant; Control, n = 3; EAE, n = 4; FA60, n = 4

Ionized calcium-binding adaptor molecule 1 (Iba1), a calcium-binding protein specifically expressed in the central nervous system, serves as a microglial marker, while CD68 is recognized as a marker for activated microglia/macrophages [18]. Thus, we utilized Iba1 and CD68 as indicators of microglial activation. Immunofluorescence staining of the intumescentia lumbalis revealed substantial infiltration of activated microglia (Iba1⁺CD68⁺) in the anterior funiculus of EAE mice compared to controls. This activation was significantly attenuated by FA treatment (Fig. 3A, B). Activated pro-inflammatory microglia secrete various cytokines, including interleukin-1beta (IL-1β), tumor necrosis factor alpha (TNF-α), and interleukin-6 (IL-6), along with inflammatory enzymes and signaling molecules such as inducible nitric oxide synthase (iNOS) and cyclooxygenase-2 (COX-2) [19]. Consistent with this profile, qRT-PCR analysis of spinal cord RNA demonstrated elevated mRNA levels of the pro-inflammatory markers IL-1β, TNF-α, and iNOS in EAE mice at the disease peak. FA treatment effectively reduced the expression of these markers (Fig. 3C–E).

### FA protection via TNF-α-induced protein 2 (Tnfaip2) suppression

#### Mechanism involving the TNF pathway

**Fig. 4:**
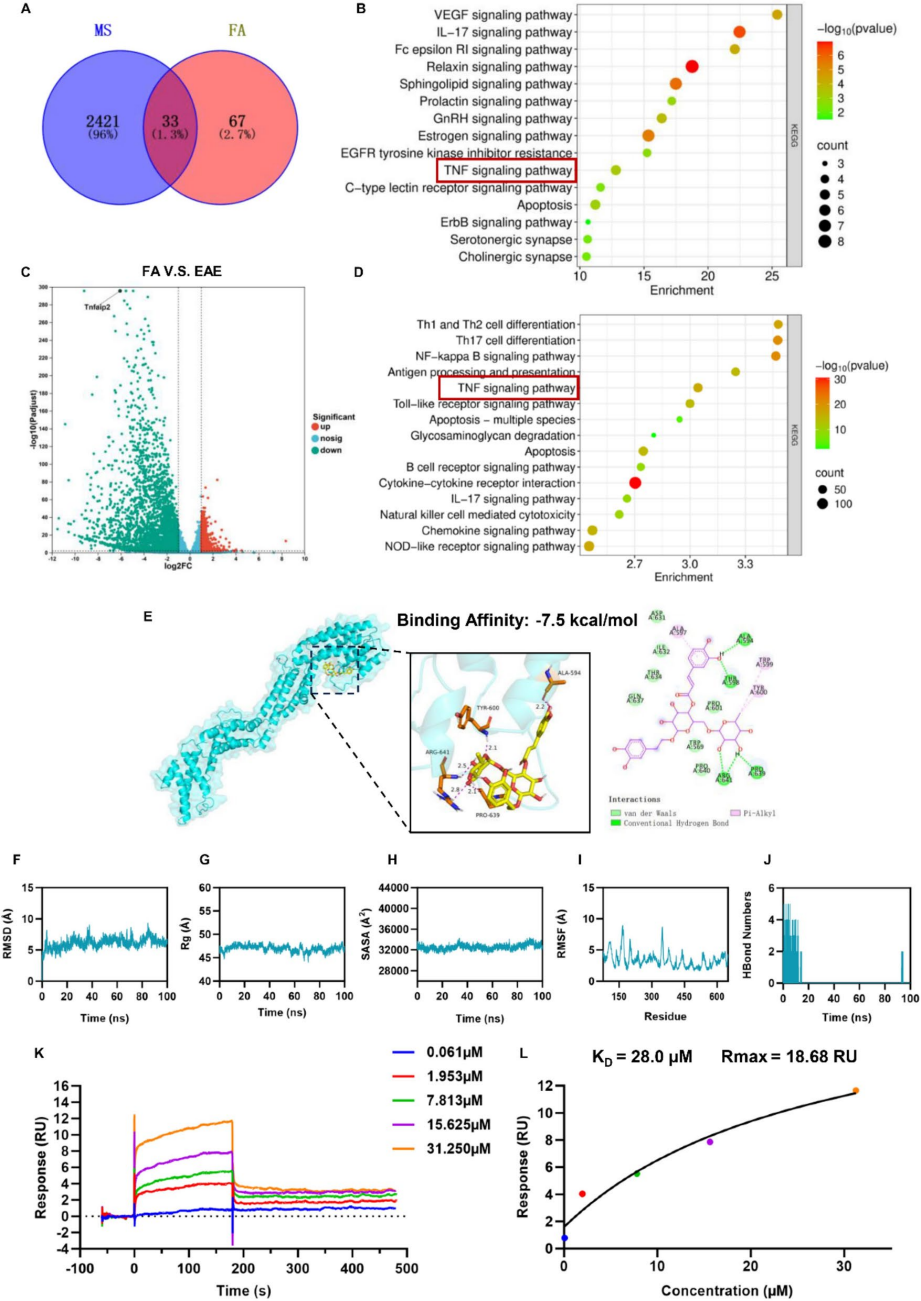
Network pharmacology and molecular docking analyses reveal the potential targets of FA for treating EAE mice. **A** Venn diagram showing the overlap between potential targets of FA and multiple sclerosis (MS)-related disease targets. **B** KEGG pathway enrichment analysis of the overlapping genes from **A**. **C** Differentially expressed genes in the spinal cord of the high-dose FA (FA60) group compared to the EAE group (n = 4 per group). **D** KEGG pathway enrichment analysis of the statistically significant genes from **C**. **E** Molecular docking model of FA with the Tnfaip2 protein. FA is represented as yellow sticks. The CARD domain of Tnfaip2 is colored blue, and interaction residues are highlighted in orange. Hydrogen bonds are shown in dark green, van der Waals forces in light green, and Pi-Alkyl interactions in pink. **F**–**J** Molecular dynamics simulation of the FA-Tnfaip2 complex: **F** root mean square deviation (RMSD), **G** radius of gyration (Rg), **H** solvent accessible surface area (SASA), **I** root mean square fluctuation (RMSF) of protein backbone atoms, and **J** number of hydrogen bonds (H-bonds) over time. **K** Surface plasmon resonance (SPR) sensorgram for FA binding to recombinant mouse Tnfaip2 protein. Response curves for increasing FA concentrations are overlaid and aligned to the injection start point. **L** Fitted binding curve for FA binding to recombinant mouse Tnfaip2 protein

To investigate the mechanism of FA, we integrated transcriptomic sequencing of EAE mouse spinal cords with network pharmacology. Kyoto encyclopedia of genes and genomes (KEGG) analysis of 33 shared targets between FA and MS (Fig. 4A, B) and of spinal cord transcriptomic data (Fig. 4C, D) both identified the TNF pathway as significantly enriched. Our transcriptomic analysis identified Tnfaip2, a known effector protein of TNF-α with significant roles in inflammation and cancer, as being significantly downregulated in the FA-treated group compared to the EAE group (Fig. 4C), suggesting it may be a functional target of FA.

#### In silico molecular docking suggests an FA-Tnfaip2 interaction

Molecular docking and molecular dynamics simulations were subsequently performed to characterize the interaction between FA and Tnfaip2. The docking results indicated that the FA ligand forms five hydrogen bonds with the amino acid residues ALA-594, TYR-600, PRO-639, and ARG-641 in the protein binding pocket, with bond distances ranging between 2 and 3 Å. Additional interactions include van der Waals forces and Pi-Alkyl interactions. The calculated binding affinity for this complex was −7.5 kcal/mol, suggesting a strong interaction between FA and Tnfaip2 (Fig. 4E).

The root mean square deviation (RMSD) serves as a key metric for evaluating the conformational stability of protein–ligand complexes by measuring the deviation of atomic positions from their initial coordinates. Lower RMSD values indicate greater conformational stability. Assessment of the RMSD profile demonstrated that the FA-Tnfaip2 complex reached equilibrium after approximately 90 ns, with fluctuations stabilizing around 6.3 Å thereafter (Fig. 4F). The radius of gyration (Rg), which reflects the overall compactness of a protein structure, exhibited modest fluctuations during the simulation, suggesting conformational adjustments of the complex (Fig. 4G). Analysis of the solvent-accessible surface area (SASA) revealed mild variations that gradually stabilized, indicating that ligand binding influences the micro-environment around the binding site and modestly alters the protein's solvent exposure (Fig. 4H). The root mean square fluctuation (RMSF), which reflects the flexibility of individual amino acid residues, remained relatively low across most of the protein structure, with values predominantly below 5 Å, indicating restricted movement in residue positions upon ligand binding (Fig. 4I). Hydrogen bonding plays a critical role in mediating ligand–protein interactions. Throughout the simulation, the number of hydrogen bonds between FA and Tnfaip2 ranged from 0 to 5, with an average of approximately 1 persistent bond, supporting the presence of favorable polar interactions (Fig. 4J). In summary, the FA-Tnfaip2 complex demonstrated stable binding throughout the simulation, supported by consistent RMSD, favorable hydrogen bonding, and limited residual fluctuation, collectively affirming a robust and well-bound interaction.

#### Surface plasmon resonance (SPR) affirms in vitro binding between FA and Tnfaip2

The binding affinity between FA and the murine Tnfaip2 protein was quantitatively measured using SPR. The analysis yielded an equilibrium dissociation constant (K_D_) of 28 µM and a maximum binding capacity (R_max_) of 18.68 RU, confirming their direct interaction in vitro (Fig. 4K, L).

### FA regulates the polarization of microglial cells

#### Tnfaip2 is highly expressed on microglia during the acute phase of EAE

**Fig. 5:**
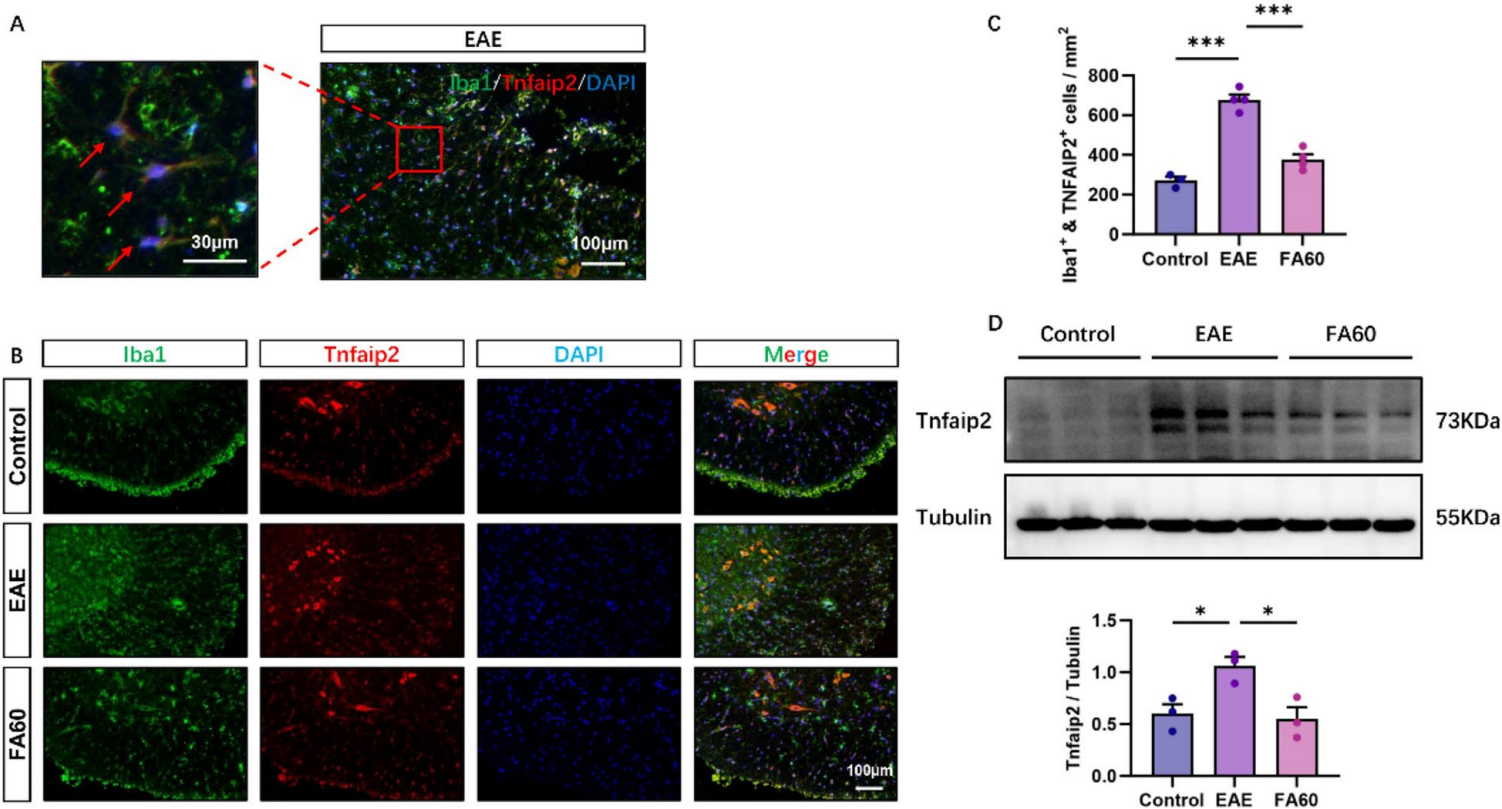
FA treatment reduces Tnfaip2 expression during the acute phase in EAE mice. **A** Immunofluorescence staining shows Tnfaip2 expression in microglia at the peak disease stage of EAE mice. **B** Representative immunofluorescence images of Tnfaip2-positive microglia in each group (Control, n = 3; EAE, n = 4; FA60, n = 4). **C** Quantification of Iba1⁺Tnfaip2⁺ microglia in the spinal cord. **D** Protein expression level of Tnfaip2 in the spinal cord at the peak disease stage (n = 3 per group). Data are presented as mean ± SEM. *p < 0.05, ***p < 0.001

Tnfaip2 is a 654-amino-acid polypeptide originally identified as a major gene induced in endothelial cells upon TNF-α stimulation. Under physiological conditions, Tnfaip2 is expressed in immune tissues such as the spleen and lymph nodes, as well as in various fetal and adult tissues [20]. Using immunofluorescence co-staining, we observed that Tnfaip2 was highly expressed in activated microglia during the acute phase of EAE (Fig. 5A–C). Consistent with this finding, Western blot analysis also confirmed the upregulation of Tnfaip2 in the acute phase of EAE, which was attenuated by FA treatment (Fig. 5D).

#### Tnfaip2 promotes pro-inflammatory microglial polarization via the NF-κB pathway

**Fig. 6:**
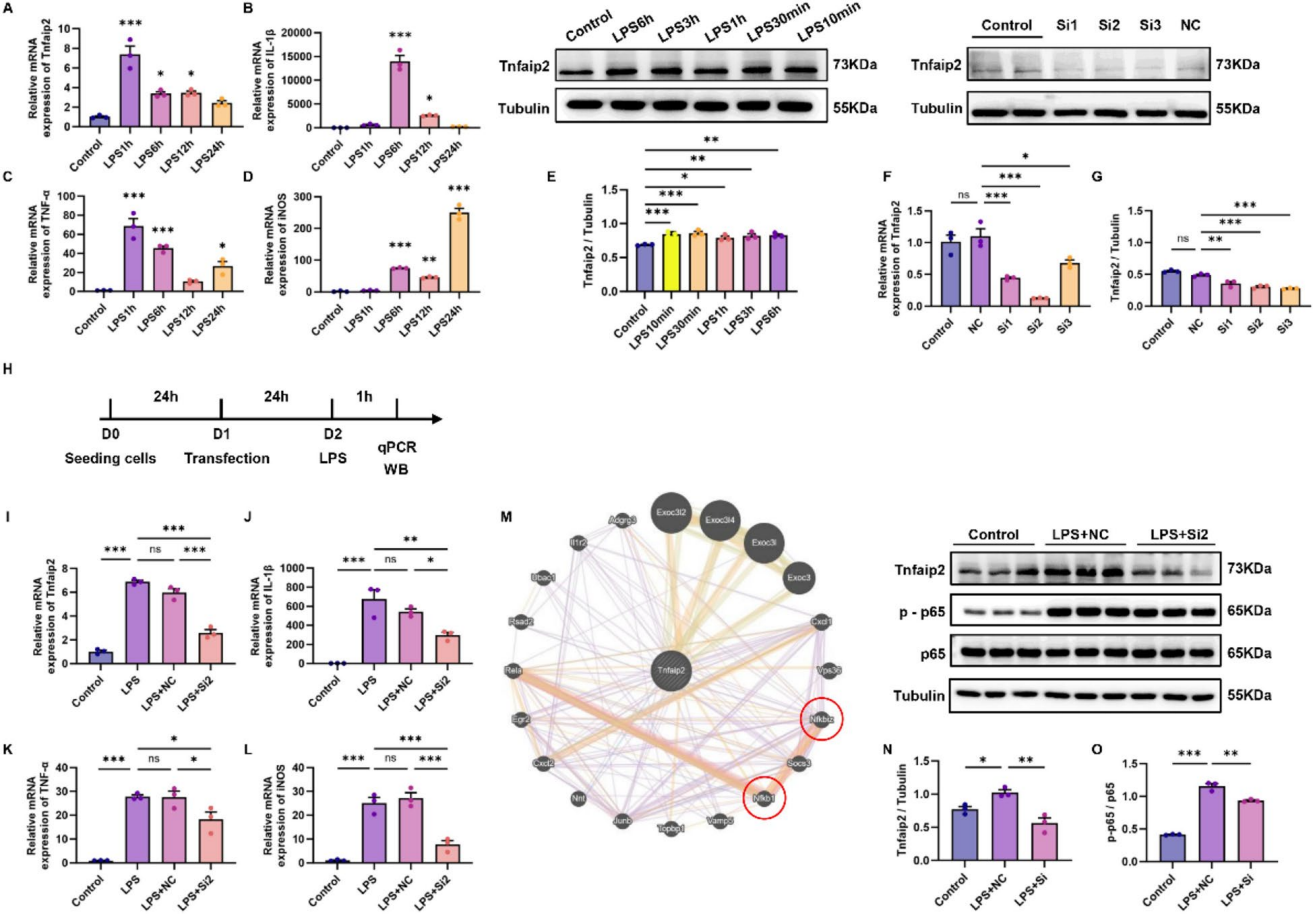
Tnfaip2 promotes pro-inflammatory polarization of microglial cells. **A**–**D** mRNA expression levels of Tnfaip2 (**A**), IL-1β (**B**), TNF-α (**C**), and iNOS (**D**) in BV2 cells stimulated with 0.5 μg/mL LPS for the indicated durations. **E** Protein expression level of Tnfaip2 in BV2 cells after LPS (0.5 μg/mL) treatment for the indicated times. **F**, **G** Efficiency of Tnfaip2 knockdown using different siRNAs, assessed at the mRNA (**F**) and protein (**G**) levels. **H** Schematic diagram of the experimental timeline for siRNA transfection and LPS stimulation in BV2 cells. **I**–**L** mRNA expression levels of Tnfaip2 (**I**), IL-1β (**J**), TNF-α (**K**), and iNOS (**L**) in BV2 cells transfected with Tnfaip2-siRNA for 24 h, followed by LPS (0.5 μg/mL) stimulation for 1 h. **M** Protein interaction network of Tnfaip2. **N** Tnfaip2 protein expression in BV2 cells treated as in **I**–**L**. **O** Ratio of phosphorylated p65 to total p65 protein in BV2 cells treated as in **I**–**L**. Data are presented as mean ± SEM. *p < 0.05, **p < 0.01, ***p < 0.001; *ns* not significant; n = 3 per group; NC = negative control

To investigate the role of Tnfaip2 in microglial polarization, we stimulated BV2 cells with LPS (0.5 µg/ml) and assessed the temporal expression profiles of Tnfaip2 and pro-inflammatory markers (Fig. 6A–D). Both transcriptional and protein levels of Tnfaip2 were elevated early after LPS stimulation and peaked rapidly (Fig. 6E), indicating that Tnfaip2 is activated during the initial inflammatory response and may be associated with pro-inflammatory polarization.

We next knocked down Tnfaip2 in BV2 cells using siRNA. 24 h post-transfection, cells were treated with LPS, and all three siRNA constructs effectively reduced Tnfaip2 expression at both RNA and protein levels (Fig. 6F, G). SiRNA-2 was selected for subsequent experiments. In Tnfaip2-knockdown cells, the LPS-induced upregulation of Tnfaip2, IL-1β, TNF-α, and iNOS was significantly attenuated compared to control cells (Fig. 6H–L), suggesting that suppression of Tnfaip2 inhibits pro-inflammatory microglial polarization.

To explore the underlying mechanism, we used the GeneMANIA database (https://genemania.org/) and identified potential physical interactions between Tnfaip2 and key NF-κB pathway components, including NFKB1 and NFKBIZ (Fig. 6M). Given the established role of NF-κB in microglial polarization, we examined its activation in Tnfaip2-knockdown cells. Phosphorylation of p65, a marker of NF-κB pathway activation, was markedly reduced in Tnfaip2-deficient cells following LPS stimulation (Fig. 6N, O), indicating that Tnfaip2 promotes pro-inflammatory polarization partly through modulating NF-κB signaling.

We stimulated BV2 cells with TNFα (100 ng/ml) for 1 h as an alternative inflammatory model to LPS. The results demonstrated that Tnfaip2 knockdown significantly suppressed TNFα-induced upregulation of IL-1β, iNOS, and IL-6 mRNA expression, and also attenuated the increase in p65 phosphorylation levels (Supplementary Fig. 1).

#### FA suppresses pro-inflammatory microglial polarization via the Tnfaip2/NF-κB pathway

**Fig. 7:**
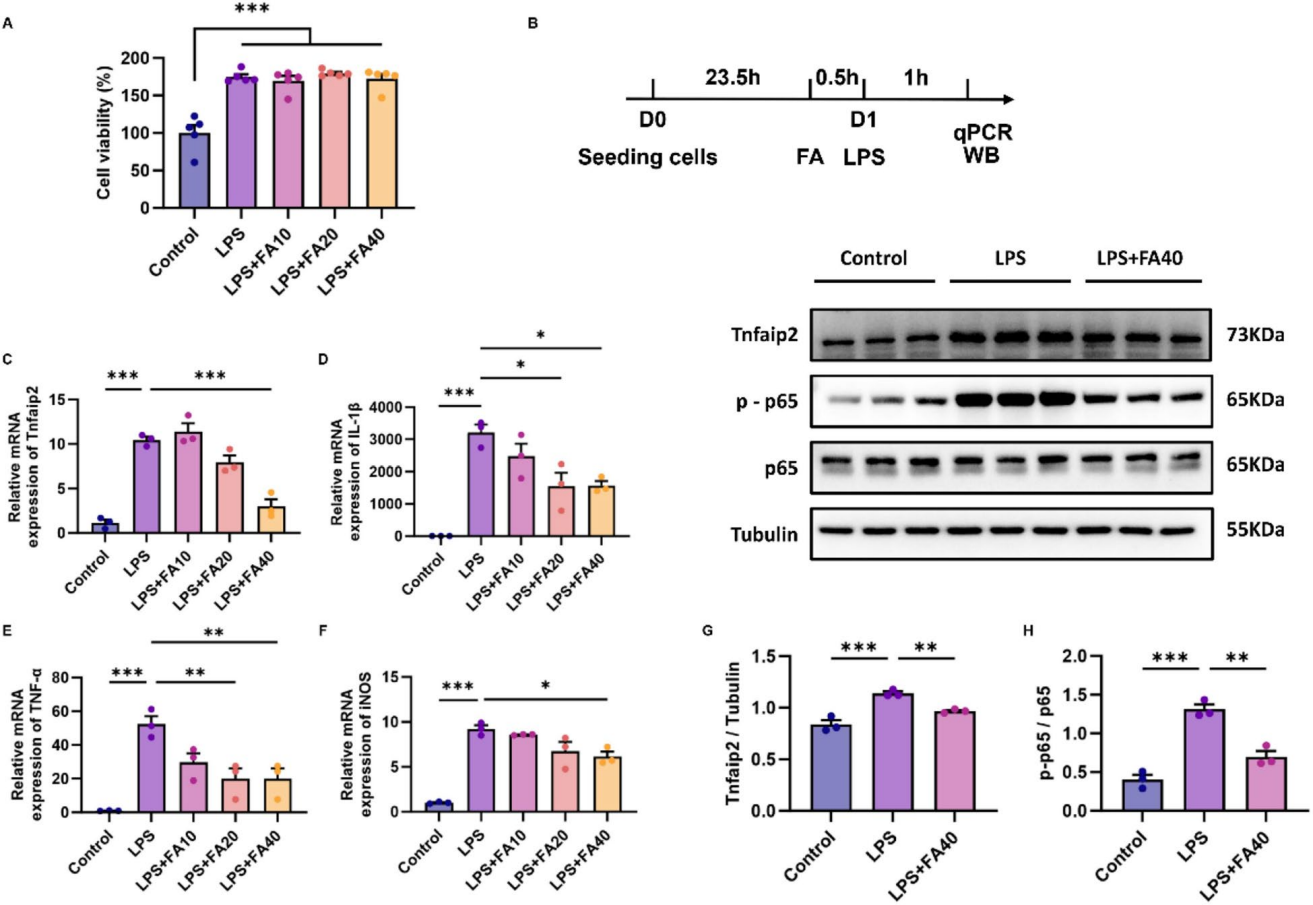
FA inhibits LPS-induced pro-inflammatory polarization of BV2 cells. **A** Cell viability was assessed by CCK-8 assay after treatment with varying concentrations of FA. **B** Schematic diagram of the experimental procedure for FA and LPS treatment in BV2 cells. **C**–**F** mRNA expression levels of Tnfaip2 (**C**), IL-1β (**D**), TNF-α (**E**), and iNOS (**F**) in BV2 cells pretreated with different concentrations of FA followed by stimulation with 0.5 μg/mL LPS for 1 h. **G** Tnfaip2 protein expression in BV2 cells pretreated with 40 μg/mL FA and then stimulated with LPS for 1 h. **H** Ratio of phosphorylated p65 to total p65 protein in BV2 cells under the same treatment as in **G**. Data are presented as mean ± SEM. *p < 0.05, **p < 0.01, ***p < 0.001; n = 3 per group

To further investigate the anti-inflammatory effect of FA, BV2 cells were pre-treated with FA at concentrations of 10, 20, and 40 µg/ml for 0.5 h before LPS (0.5 µg/ml) stimulation. Cell counting kit-8 (CCK-8) assays confirmed that none of these concentrations exhibited cytotoxicity (Fig. 7A). The mRNA levels of Tnfaip2, IL-1β, TNF-α, and iNOS were measured 1 h after LPS stimulation. Results showed that FA dose-dependently inhibited the expression of these pro-inflammatory markers, with 40 µg/ml significantly attenuating LPS-induced microglial polarization toward a pro-inflammatory phenotype (Fig. 7B–F). Western blot analysis further confirmed that 40 µg/ml FA suppressed activation of the Tnfaip2/NF-κB pathway (Fig. 7G, H).

**Fig. 8:**
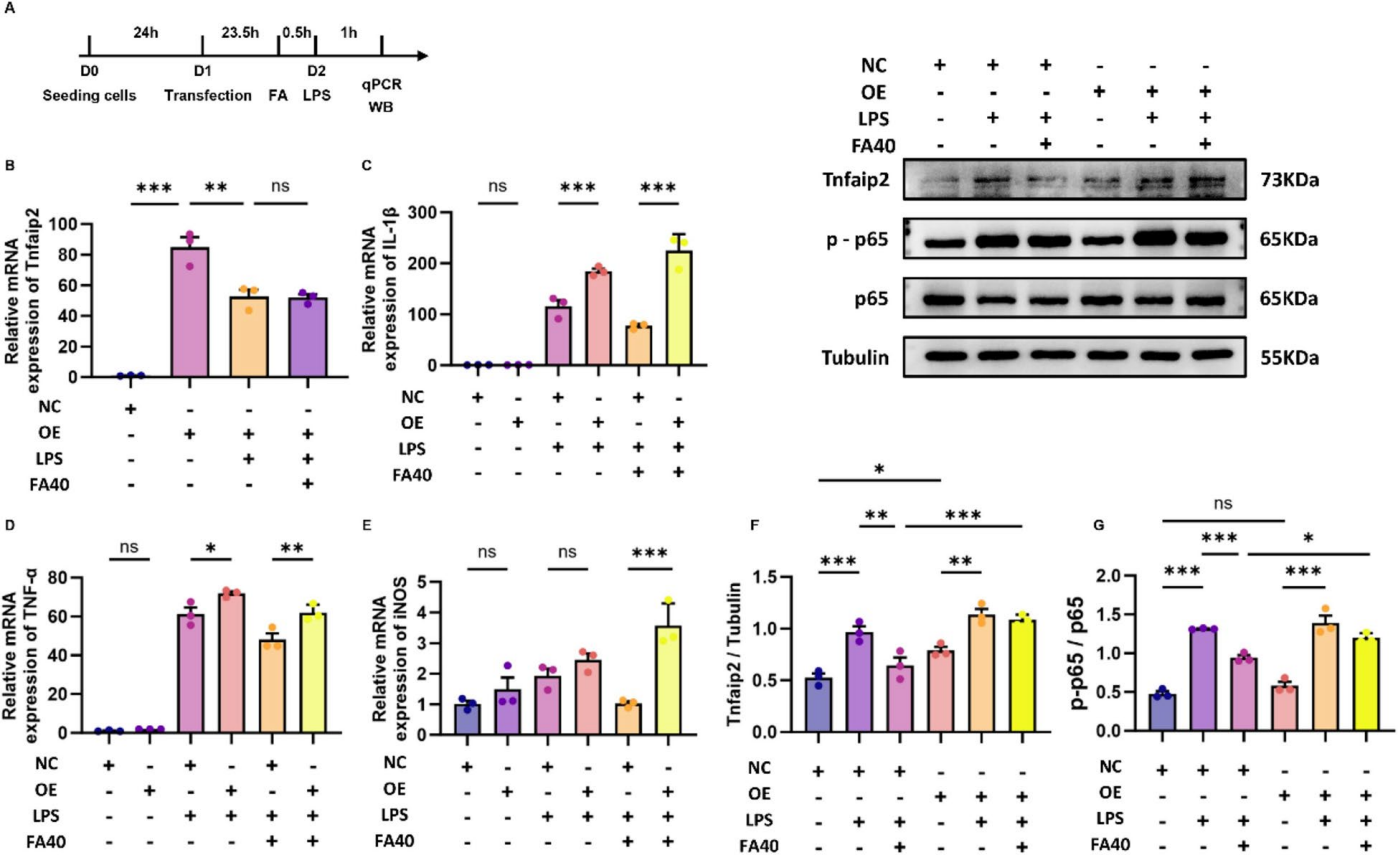
Overexpression of Tnfaip2 in BV2 cells attenuates the inhibitory effects of FA. **A** Schematic diagram of the experimental procedure for Tnfaip2 overexpression and subsequent treatment with FA and LPS. **B**–**E** mRNA expression levels of Tnfaip2 (**B**), IL-1β (**C**), TNF-α (**D**), and iNOS (**E**) in BV2 cells overexpressing Tnfaip2 for 24 h, followed by treatment with 40 μg/mL FA and 0.5 μg/mL LPS. **F** Tnfaip2 protein expression in BV2 cells under the same treatment conditions as in **B**–**E**. **G** Ratio of phosphorylated p65 to total p65 protein in BV2 cells under the same treatment as in **B**–**E**. Data are presented as mean ± SEM. *p < 0.05, **p < 0.01, ***p < 0.001; *ns* not significant; n = 3 per group; OE = overexpression

To determine whether Tnfaip2 is essential for the anti-inflammatory effect of FA, we overexpressed Tnfaip2 in BV2 cells before FA treatment. Tnfaip2 overexpression exacerbated the LPS-induced inflammatory response and reversed the inhibitory effects of FA, as evidenced by increased expression of IL-1β, TNF-α, and iNOS (Fig. 8A–E), along with enhanced NF-κB activation (Fig. 8F, G). Notably, in the absence of inflammatory stimulation, Tnfaip2 overexpression alone did not activate the NF-κB pathway or upregulate pro-inflammatory markers. In addition, Tnfaip2 knockdown alone did not induce changes in pro-inflammatory markers or phosphorylation of p65 (Supplementary Fig. 1).

## Discussion

This study systematically investigated the therapeutic effects of FA on EAE and its underlying molecular mechanisms through both in vivo and in vitro experiments. The results demonstrated that FA significantly alleviated behavioral symptoms, CNS inflammation, and demyelination in EAE mice, while also suppressing peripheral Th1 cell differentiation. Mechanistically, this study reveals for the first time that FA may exert its therapeutic effects by targeting Tnfaip2, thereby blocking hyperactivation of the NF-κB signaling pathway and inhibiting pro-inflammatory microglial polarization. These findings not only elucidate a novel mechanism by which FA may treat multiple sclerosis but also provide the first exploration of the role of Tnfaip2 in microglial polarization, offering a strong theoretical basis for developing Tnfaip2 as a new therapeutic target for MS.

When the blood–brain barrier is disrupted, large numbers of highly pro-inflammatory and autoreactive leukocytes infiltrate the central nervous system, leading to demyelination, oligodendrocyte death, axonal injury, and even neuronal damage. Parallel to these autoimmune processes is the persistent activation of resident macrophages/microglia. A hallmark of acute MS lesions is the abrupt appearance of abundant activated microglia and macrophages, which outnumber lymphocytes by at least 10- to 20-fold [21–23]. Activated microglia continuously release inflammatory cytokines and reactive oxygen species (ROS), further attacking neurons and oligodendrocytes and resulting in neuronal injury and demyelination. Multiple studies have confirmed that suppressing pro-inflammatory microglial activation can alleviate symptoms in EAE mice [24–28]. In recent years, Bruton tyrosine kinase (BTK) inhibitors have garnered significant attention for their ability to cross the blood–brain barrier and directly target microglia within the central nervous system, thereby offering an innovative therapeutic mechanism for multiple sclerosis [25, 29]. Consequently, "targeting microglia" has emerged as a highly promising direction in MS research. Unlike systemic immunosuppression, modulating the functional state of microglia aims to reshape the immune microenvironment from within the CNS, potentially achieving greater specificity and fewer adverse effects.

Forsythoside A, also referred to as Forsythiaside A, is a primary active compound derived from *Forsythia suspensa* (Thunb.) Vahl plant. FA exhibits a range of beneficial pharmacological properties, including anti-inflammatory, antiviral, neuroprotective, antioxidant, hepatoprotective, and antibacterial activities [30–33]. Previous studies have reported that oral administration of FA reduces the number of microglia and astrocytes in gerbils following transient global cerebral ischemia [16]. In vitro evidence further confirms that FA suppresses LPS-induced inflammatory responses in both BV2 microglial cells and primary microglia [34]. In the present study, we demonstrate that FA delays disease onset and alleviates clinical severity in EAE mice by inhibiting pro-inflammatory polarization of microglia. Further investigation revealed that FA mediates this anti-inflammatory effect by targeting Tnfaip2.

**Scheme 1:**
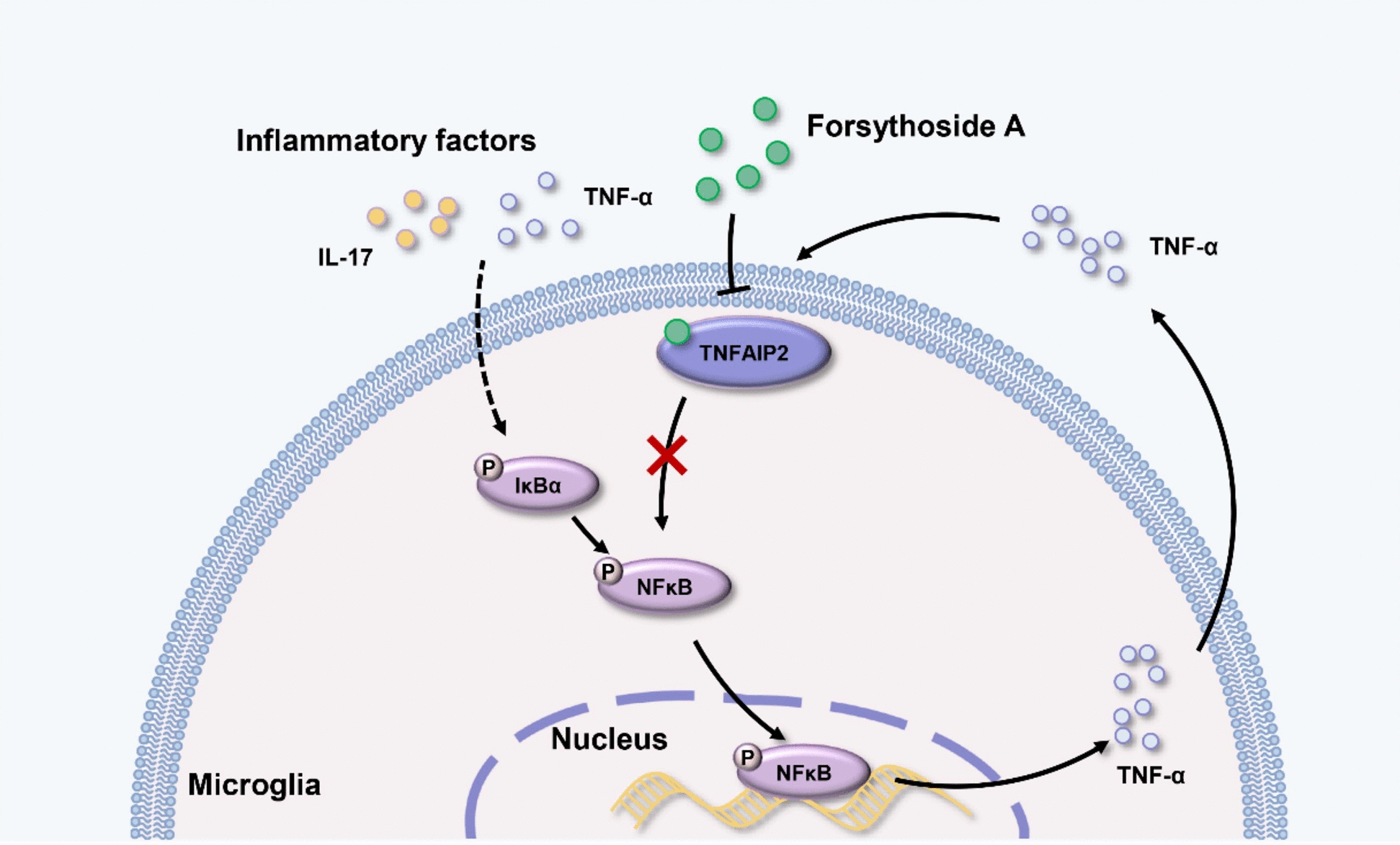
In the EAE model, pathogenic T helper cell subsets secrete pro-inflammatory cytokines (e.g., TNF-α, IFN-γ, IL-17), which trigger pro-inflammatory activation of microglia and macrophages. TNF-α simultaneously induces Tnfaip2, which sustains NF-κB signaling to promote further microglial polarization towards a pro-inflammatory phenotype. FA suppresses microglial pro-inflammatory polarization by inhibiting Tnfaip2, thereby exerting neuroprotective effects

TNF-α-induced proteins (TNFAIPs) have gained increasing research interest in recent years. Tnfaip2, also known as B94 or MSec, is a protein induced by TNF-α [35]. Its mRNA is highly expressed in immune organs such as the spleen and lymph nodes, as well as in fetal kidney, fetal and adult lung, and placenta [20]. Tnfaip2 expression is also enriched in endothelial cells, granulocyte-monocyte progenitors, peripheral blood monocytes, intestinal M cells, dendritic cells, macrophages, and mature sperm [36]. Initially identified as an angiogenic factor, Tnfaip2 has recently attracted attention for its roles in tumor proliferation, metastasis, drug resistance, and immune regulation in cancer progression [37–39]. However, growing evidence suggests that Tnfaip2 also plays important roles in inflammation regulation [40–42], lipid metabolism, and oxidative stress [43,44]. A pivotal connection to neuroinflammatory disease was established by a whole-exome sequencing study in familial MS. This study, analyzing gene variants within the TNF-α signaling pathway across 19 families with at least two MS patients, identified a particularly significant association between the Tnfaip2 gene variant rs1132339 and autoimmune diseases, including MS [45]. This finding extended the implications of Tnfaip2 polymorphisms from the field of oncology to autoimmune disorders, suggesting a broader role for this gene in modulating genetic susceptibility to inflammatory and immune-mediated diseases. Our study builds upon this foundation by providing the first direct evidence for a critical role of Tnfaip2 in the pathogenesis of EAE and in the regulation of microglial polarization. We show that inflammatory stimuli not only induce TNF-α production but also potently upregulate Tnfaip2 in microglia. Importantly, our study reveals that Tnfaip2 acts as a unique "amplifier" in pro-inflammatory polarization of microglia. It enhances TNF-α expression via the NF-κB pathway, thereby promoting pro-inflammatory microglial polarization and exacerbating neuroinflammation (Scheme 1). Mechanistically, Tnfaip2 has been reported to sustain NF-κB signaling by competitively binding KEAP1 and protecting IKKβ from ubiquitin-dependent degradation [39]. Furthermore, this relationship is bidirectional, as Tnfaip2 itself is a transcriptional target of NF-κB. Studies have demonstrated that p65 directly binds to an NF-κB response element within the Tnfaip2 promoter (located at −3869 to −3860 bp) [46]. Upon inflammatory stimulation, activated NF-κB drives Tnfaip2 transcription, and the newly synthesized Tnfaip2 protein in turn sustains NF-κB signaling by protecting IKKβ from degradation, thereby establishing a positive feedback loop that amplifies and prolongs the inflammatory response. Notably, Tnfaip2 only serves an "amplifying" role in inflammation-elevating Tnfaip2 expression alone does not initiate inflammatory responses. These findings identify Tnfaip2 as a key node in a pathogenic amplification loop and a promising therapeutic target.

A well-known "TNF-α paradox" exists in its biological functions. In clinical practice, although anti-TNF-α antagonists (such as Infliximab and Lenercept) are effective treatments for diseases like rheumatoid arthritis [47], they can induce or worsen the clinical symptoms of MS [48,49]. This suggests that TNF-α may play a dual role in the immune and nervous systems—for instance, it might also participate in immune regulation or remyelination under certain conditions [50]. Therefore, identifying more specific downstream targets has become an important research direction. Our study identified a potentially more specific downstream target—Tnfaip2, and demonstrated that FA disrupts the inflammatory cascade by inhibiting Tnfaip2, thereby exerting therapeutic effects. These findings provide a rationale for the use of FA in treating MS. Although the SPR analysis revealed a moderate binding affinity (K_D_ = 28 µM) between FA and Tnfaip2, this value falls within the range commonly observed for many natural products with proven in vivo efficacy [51,52]. It is important to note that moderate binding affinity does not preclude functional relevance, particularly when combined with sustained drug exposure or tissue-specific enrichment. We acknowledge the absence of direct CNS exposure data in the current study. However, previous investigations have demonstrated that FA can cross the blood–brain barrier via both passive diffusion and active transport [13]. Moreover, in the EAE model, the integrity of the blood–brain barrier is compromised during the acute phase, which may facilitate enhanced accumulation of FA in inflamed CNS lesions [53]. Furthermore, our transcriptomic and network pharmacology analyses identified multiple pathways enriched upon FA treatment, including the TNF pathway, NF-κB signaling, and oxidative stress responses. These findings suggest that FA may exert its neuroprotective effects through a multi-target mechanism, consistent with the polypharmacology of many natural products [54]. Tnfaip2 represents a newly identified node within this network, but it is likely that FA also modulates other targets such as Nrf2, TLR4, or JAK/STAT, as previously reported [34, 55,56].

In the present study, FA treatment resulted in two notable immunological changes: a reduction in peripheral Th1 cell frequencies and an inhibition of central microglial pro-inflammatory polarization. However, our experimental design does not allow us to distinguish whether these are independent parallel events, or whether one is causally dependent on the other. FA may exert its multidimensional immunomodulatory effects through the following mechanisms: (1) Direct peripheral action: FA or its metabolites may directly modulate T cell differentiation in peripheral immune organs such as lymph nodes and spleen, thereby reducing the generation of pathogenic Th1 cells. (2) Indirect CNS-periphery crosstalk: By suppressing microglial activation within the CNS, FA reduces local neuroinflammation. A stabilized CNS microenvironment may, in turn, feedback to modulate peripheral immune responses through altered chemokine secretion or functional modulation of infiltrating immune cells. (3) Shared signaling pathways: NF-κB serves as a critical signaling node in both peripheral T cell activation and central microglial polarization. By targeting Tnfaip2 to inhibit NF-κB signaling, FA may exert concurrent anti-inflammatory effects in both compartments. Previous reports have demonstrated that FA ameliorates influenza A virus infection by regulating Th1/Th2 and Th17/Treg balance [12, 32, 57]. The precise mechanisms underlying FA-mediated peripheral immunomodulation warrant further investigation.

There are some limitations in our study. First, due to the low incidence of MS and the difficulty in obtaining clinical samples, we did not validate whether Tnfaip2 is elevated in patients with acute-phase MS. Second, we lack genetic loss-of-function models to definitively establish the causal role of Tnfaip2 in mediating the therapeutic effects of FA. Our conclusions are primarily derived from in vitro Tnfaip2 knockdown/overexpression experiments and in vivo correlation analyses. While these data strongly support a close association between FA efficacy and Tnfaip2 downregulation, they cannot fully rule out contributions from other pathways or confirm that Tnfaip2 is the sole mediator. Generation of microglia-specific Tnfaip2 knockout mice will be essential to directly validate its role in EAE pathogenesis and FA therapeutic response in future studies. Third, while the BV2 microglial cell line is a widely used and valuable tool for studying microglial biology, it may not fully recapitulate the characteristics of primary microglia. Previous studies have reported differences between BV2 cells and primary microglia in terms of their inflammatory responses, including cytokine secretion profiles, receptor expression, and activation thresholds [58,59]. Therefore, the anti-inflammatory effects of FA observed in BV2 cells should be interpreted with caution. Future studies using primary microglia will be necessary to confirm our findings and to better understand the role of the Tnfaip2/NF-κB pathway in microglial polarization under more physiologically relevant conditions.

## Conclusions

In summary, our study demonstrates the therapeutic potential of FA as a natural compound. More importantly, it identifies the Tnfaip2/NF-κB pathway as a promising novel target for intervention in neuroinflammatory diseases such as multiple sclerosis. While further validation using Tnfaip2-deficient animal models is warranted, this study provides a strong preclinical rationale for targeting this pathway.

## Materials and methods

### Animals and the EAE mouse model

Female C57BL/6 mice, aged 8 weeks and weighing 16–18 g, were acquired from GemPharmatech Co., Ltd. (Beijing, China). Housing was provided under specific pathogen-free conditions at a constant temperature of 22 °C with a 12-h light/dark cycle. Mice were accommodated five per cage and allowed free access to food and water. The entire animal study protocol was approved by the Animal Ethics Committee of Tianjin Medical University (Approval No. TMUaMEC2024022) and was performed in compliance with the ARRIVE guidelines [60]. For EAE induction, the myelin oligodendrocyte glycoprotein peptide fragment 35–55 (MOG_35–55_; MEVGWYRSPFSRVVHLYRNGK; NJPeptide Ltd., China) was dissolved in phosphate-buffered saline (PBS) and emulsified in an equal volume of complete Freund's adjuvant (CFA; Sigma-Aldrich, USA) containing Mycobacterium tuberculosis (Difco, USA). Pertussis toxin (List Biological, USA) was diluted in sterile saline. Each mouse in the experimental groups received a subcutaneous injection of 0.1 mL of the MOG_35–55_/CFA emulsion at two sites in the dorsal hind footpads and at the base of the tail. Additionally, all mice were administered an intraperitoneal injection of 0.25 mL pertussis toxin on the day of immunization, followed by a second identical dose (0.25 mL, i.p.) 48 h later [61]. Behavioral signs of EAE were assessed daily by monitoring body weight changes and applying the following clinical scoring scale: 0, no clinical signs of disease; 0.5, partial tail limpness; 1, flaccid tail (tail paralysis); 2, uncoordinated gait with mild hind limb weakness; 2.5, paralysis of one hind limb; 3, paraplegia (complete paralysis of both hind limbs); 3.5, hind limb paralysis with weakened forelimb strength; 4, tetraplegia (paralysis of all four limbs); 5, moribund state or death [62].

### Drug treatments

On day 7 post-immunization, mice were randomly assigned to receive once-daily oral gavage of either vehicle (ddH₂O, control) or FA (Biopurify Co., Ltd.; China) at two different dosages: a low-dose group (20 mg/kg) and a high-dose group (60 mg/kg).

### Histopathological analysis

At the peak of disease (day 15 post-immunization), mice from each group were anesthetized with 2–3% isoflurane and transcardially perfused with 4% paraformaldehyde. The spinal cords were then carefully dissected out, fixed, and embedded in paraffin. Subsequently, transverse sections were cut at a thickness of 5 μm. The sections were stained with H&E or LFB. All stained sections were examined and imaged using a Nikon digital light microscope (Tokyo, Japan). Quantitative analysis was performed using ImageJ 6.0 software. For H&E-stained sections, the number of inflammatory cells in each image was counted and normalized to the tissue area, with the final results expressed as "the number of inflammatory cells per mm^2^" [63]. For LFB staining, the positively stained pixel area in each image was calculated and divided by the total pixel area of the field of view. The myelin-positive area was subsequently expressed as a percentage (positive pixel area/total pixel area × 100%) [64].

### Immunofluorescence staining

At the disease peak, mice from all groups were anesthetized with 2–3% isoflurane. Spinal cord tissues were subsequently dehydrated in a graded sucrose series (15% and 30%), embedded in OCT compound, and rapidly frozen in liquid nitrogen. Transverse sections of the lumbar spinal cord were cut at a thickness of 8 μm using a cryostat (Leica CM1950, Germany). For each animal, three non‑consecutive sections (spaced at least 50 μm apart) were selected for analysis. Immunofluorescence staining was performed as previously described [64]. Briefly, sections were permeabilized with 0.5% Triton X‑100, blocked with 5% bovine serum albumin (BSA), and incubated overnight at 4 °C with primary antibodies. After three washes with PBS, sections were incubated with corresponding fluorescent dye‑conjugated secondary antibodies for 1 h at 37 °C, followed by nuclear counterstaining with DAPI. Stained sections were imaged using a Nikon digital fluorescence microscope (Tokyo, Japan). For each section, images were captured from the bilateral anterior funiculus of the lumbar spinal cord. Exposure settings were kept identical for all samples within the same experimental batch. The following primary antibodies were used: anti-MBP (1:100; Boster, BA0094, China), anti-Iba1 (1:100; Abcam, AB5076, USA), anti-CD68 (1:100; Abclonal, A23205, China), and anti-Tnfaip2 (1:100; Abcam, AB196659, USA). Secondary antibodies included donkey anti-rabbit (1:100; Abmart, M213869, China) and donkey anti-goat (1:100; Abmart, M213267, China).

Quantification: All quantifications were performed in a blinded manner by an investigator unaware of the experimental groups. For MBP staining, the percentage of MBP‑positive area was quantified using ImageJ software. A uniform color threshold was applied to each image to separate the positive signal from the background, and the positive pixel area was divided by the total pixel area of the field. The values from all fields and sections were averaged to obtain a single value per mouse. Results are expressed as the mean percentage of MBP‑positive area per animal. For Iba1 and CD68/Tnfaip2 co‑staining, the number of Iba1⁺CD68⁺ or Iba1⁺Tnfaip2⁺ cells was counted manually in ImageJ using the merged images and DAPI staining to identify nuclei. The regions of interest (ROIs) for Iba1‑ and CD68/Tnfaip2‑positive signals were first generated by applying a uniform threshold; the overlapping region was then used to count double‑positive cells. Cell counts were normalized to the tissue area and expressed as the number of Iba1⁺CD68⁺ or Iba1⁺Tnfaip2⁺ cells per mm^2^. For each animal, the average value from all analyzed sections and fields was calculated and used for subsequent statistical analysis.

### Flow cytometry

At the peak of the disease, spleens were harvested without PBS perfusion. Single-cell suspensions were prepared by mechanically disrupting the spleens through a 40-μm cell strainer. Red blood cells were lysed using a commercial lysis buffer (Solarbio, China). For intracellular cytokine staining, splenocytes were stimulated for 6 h with a mixture of phorbol 12-myristate 13-acetate (PMA), ionomycin, Brefeldin A (BFA), and Monensin. The stimulated cells were then surface-stained with anti-CD3-FITC (clone 145-2C11) and anti-CD4-PerCP-Cy5.5 (clone GK1.5). Following fixation and permeabilization, cells were intracellularly stained with anti-IFN-γ-PE (clone XMG1.2), anti-IL-4-APC (clone 11B11), and anti-IL-17A-PE (clone 17B7). Staining procedures were performed using mouse Th1/Th2 and Th17 staining kits (Multi Sciences, China) according to the manufacturer's instructions. Data were acquired on a FACSAria flow cytometer (BD Biosciences, USA) and analyzed using FlowJo software. The gating strategy is detailed in Supplementary Fig. 2.

### RNA sequencing and functional enrichment analysis

RNA sequencing services were provided by Majorbio (Shanghai, China). Total RNA was isolated from PBS-perfused, snap-frozen spinal cords of EAE and high-dose FA-treated mice using the QIAzol Lysis Reagent (Qiagen, Germany). RNA quality was confirmed (Nanodrop 2000; Agilent 5300) before library preparation with the NEBNext^®^ Ultra^™^ RNA Library Prep Kit for Illumina^®^. After purification and size selection (250–300 bp), libraries were amplified and quality-checked before 150 bp paired-end sequencing on an Illumina Novaseq platform. Data analysis involved transcript assembly and FPKM calculation using StringTie (version 1.3.3 b). Differential expression analysis identified genes with a |fold change|> 2 and an adjusted p < 0.05. These genes were subjected to heatmap generation and KEGG pathway enrichment analysis. Among them, the FDR of KEGG enrichment analysis is less than or equal to 0.05, all of which meet the requirements and statistical significance of genes significantly enriched.

### BV2 cell culture

The murine BV2 microglial cell line was obtained from Origincell (Shanghai, China). Cells were seeded at a density of 3 × 10^5^ cells per mL and maintained in Dulbecco's modified Eagle's medium (DMEM) supplemented with 10% fetal bovine serum (FBS) and 1% penicillin–streptomycin in 10-cm culture dishes at 37 °C under a humidified atmosphere of 5% CO₂.

### Real-time quantitative polymerase chain reaction (qRT-PCR)

Total RNA was extracted from mouse spinal cord tissues or BV2 cells using TRIzol^™^ Reagent (Ambion, USA) following the manufacturer's protocol. Subsequently, RNA was reverse-transcribed into complementary DNA (cDNA) using the SuperScript^™^ One-Step RT-PCR Kit (Invitrogen, USA). qRT-PCR was performed on an Applied Biosystems real-time PCR detection system using iTaq^™^ Universal SYBR^®^ Green Supermix (Bio-Rad, USA). Each sample was independently amplified in at least three technical replicates. Relative gene expression was calculated using the 2^(–ΔΔCt) method. The primer sequences used are listed in Table 1.

**Table 1:** Primers used for quantitative real-time PCR

Gene Mus	Primer	Sequence, 5′−3′
IL-1β	F	GCTGAAAGCTCTCCACCTCA
	R	AGGCCACAGGTATTTTGTCG
iNOS	F	CAAGCACCTTGGAAGAGGAG
	R	AAGGCCAAACACAGCATACC
TNF-α	F	GAGGCCAAGCCCTGGTATG
	R	CGGGCCGATTGATCTCAGC
IL-6	F	CCGGAGAGGAGACTTCACAG
	R	TCTGCAAGTGCATCATCGTT
Tnfaip2	F	GGGTCGCACCATGAAGGAG
	R	GAAGTGGTAGTGGTAGCTTTCG
GAPDH	F	AGCCCAAGATGCCCTTCAGT
	R	CCGTGTTCCTACCCCCAATG

### Western blot analysis

Total protein was extracted from spinal cord tissues or BV2 cells using RIPA lysis buffer supplemented with phosphatase inhibitors, benzonase nuclease, and phenylmethylsulfonyl fluoride (PMSF). The lysates were centrifuged, and the supernatants were collected for protein concentration determination using a bicinchoninic acid (BCA) assay. Protein samples were then separated by sodium dodecyl sulfate–polyacrylamide gel electrophoresis (SDS-PAGE) and transferred onto polyvinylidene fluoride (PVDF) membranes. After blocking with 5% non-fat milk, the membranes were incubated overnight at 4 °C with specific primary antibodies. Following three washes with TBS containing 0.1% Tween-20 (TBST), the membranes were incubated with corresponding horseradish peroxidase (HRP)-conjugated secondary antibodies for 1 h at room temperature. Protein bands were visualized using the Easy Blot ECL Kit (Biosharp, China) and quantified with ImageJ software. The following primary antibodies were used: p65 (1:1000; CST, 8242, USA), phospho-p65 (1:1000; CST, 3033, USA), Tnfaip2 (1:1000; Abcam, AB196659, USA), and Tubulin (1:5000; CST, 2146, USA).

### Cell viability assay

BV2 microglial cells were seeded in 96-well plates at a density of 1 × 10^5^ cells per mL and cultured overnight. Subsequently, the cells were treated with FA (10 µg/ml; 20 µg/ml; 40 µg/ml) and lipopolysaccharide (LPS; 0.5 μg/ml). Cell viability was assessed using a CCK-8 (Biosharp, China) according to the manufacturer's instructions. Briefly, after treatments, 10 µL of CCK-8 solution was added to each well and incubated for 1 h at 37 °C. The absorbance of the resulting mixture was then measured at 450 nm using a microplate reader [41].

### Tnfaip2 siRNA transfections

BV2 cells were seeded in 6-well plates and transfected with either Tnfaip2-specific siRNA (300 nM) or a negative control siRNA using the CALNP^™^ RNAi in vitro Transfection Reagent (D-Nano Therapeutics, China), following the manufacturer's protocol. For each well, the transfection complex was prepared by mixing 3 µL of siRNA with 21 µL of reagent A and 6 µL of reagent B. Before transfection, the culture medium was replaced with serum-free DMEM, and the mixture was then added to the cells. After 24 h of incubation, transfection efficiency was assessed by measuring Tnfaip2 expression levels using both Western blot analysis and qRT-PCR. The siRNA sequences used are listed in Table 2.

**Table 2:** Sequences of siRNA used in RNA interference

Gene Mus	Sequence	Sequence, 5′−3′
mTNFAIP2 siRNA-1	Sense	GCAAGAGAUUUCUUGUAAATT
	Antisense	UUUACAAGAAAUCUCUUGCTT
mTNFAIP2 siRNA-2	Sense	GCUUGUUGAUGACAUUAAUTT
	Antisense	AUUAAUGUCAUCAACAAGCTT
mTNFAIP2 siRNA-3	Sense	CAAUCUACCAAGCAGUGAATT
	Antisense	UUCACUGCUUGGUAGAUUGTT
siRNA-NC	Sense	UUCUCCGAACGUGUCACGUTT
	Antisense	ACGUGACACGUUCGGAGAATT

### Tnfaip2 mRNA transfections

To overexpress Tnfaip2, BV2 cells seeded in 6-well plates were transfected with in vitro-transcribed mRNA encoding the mouse Tnfaip2 protein. A corresponding non-targeting mRNA was used as the negative control. Transfection was performed using the CALNP^™^ mRNA in vitro Transfection Reagent (D-Nano Therapeutics, China) according to the manufacturer's instructions, following a procedure analogous to that described in the siRNA transfections section. At 24 h post-transfection, the efficiency of Tnfaip2 overexpression was confirmed by Western blot analysis and qRT-PCR, respectively.

### Network pharmacology-based analysis of potential mechanisms of FA in MS treatment

The potential mechanisms of FA for treating MS were investigated using a network pharmacology approach. First, MS-associated targets were obtained from the GeneCards database (https://www.genecards.org/) using "multiple sclerosis" as a keyword. From the 17,127 initially retrieved genes, 2,454 genes with a relevance score greater than 10 were selected. Subsequently, the canonical SMILES of FA was retrieved from the PubChem database (https://pubchem.ncbi.nlm.nih.gov/) [65]. This SMILES string was then submitted to the SwissTargetPrediction platform (https://www.swisstargetprediction.ch/) to identify potential protein targets, from which the top 100 prediction results were collected [66]. The overlapping targets between FA and MS were identified by submitting the respective gene lists to the Venny 2.1 online tool (https://bioinfogp.cnb.csic.es/tools/venny/), which revealed 33 common targets. Finally, KEGG pathway enrichment analysis for these 33 common targets was performed using the DAVID bioinformatics database (https://david.ncifcrf.gov/).

### Molecular docking and molecular dynamics simulation

Molecular docking is a computational method used to predict the interaction between a small molecule and a protein target of known three-dimensional structure [67]. The protein sequence of Tnfaip2 was retrieved from the UniProtKB database (https://www.uniprot.org/). This sequence was then submitted to the SWISS-MODEL workspace (https://swissmodel.expasy.org/) to generate a three-dimensional homology model, from which the optimal structure was selected for subsequent analysis. The three-dimensional chemical structure of FA was downloaded from the PubChem database (https://pubchem.ncbi.nlm.nih.gov/). Using Discovery Studio 2019 and PyMOL 3.1, the protein structure underwent energy minimization and preprocessing. Molecular docking was performed with AutoDock Vina 2.1.6, conducting 50 independent docking runs. The conformation with the most favorable binding affinity and the highest frequency of occurrence among the replicates was selected as the final docking pose. This result was visualized and analyzed using both PyMOL 3.1 and Discovery Studio 2019 [68].

Molecular dynamics (MD) simulation is a computational technique used to model the dynamic behavior and trajectories of small molecules under conditions that mimic the physiological environment. 100 ns molecular MD of the complexes was performed using the Gromacs2022 software. Charmm 36 [69] was chosen as the protein force field, Gaff2 was chosen as the ligand force field, the TIP3P water model was chosen to add solvents to the protein–ligand system, and to create a water box with a periodic boundary of 1.2 nm. The particle grid Ewald (PME) and Verlet algorithms are used to deal with electrostatic interactions, respectively. Then, 100,000 steps of isothermal isovolumetric ensemble equilibrium and isothermal isobaric ensemble equilibrium were simulated with a coupling constant of 0.1 ps and a duration of 100 ps. Both van der Waals and Coulomb interactions are calculated using 1.0 nm cutoff values. Finally, the system was simulated using Gromacs 2022 at constant temperature (310 K) and constant pressure (1 bar) for a total of 100 ns [70].

### Surface plasmon resonance assay

SPR is a label-free optical technique widely employed for the real-time analysis of biomolecular interactions [71]. SPR was employed to assess the interaction between FA and Tnfaip2 protein using a Biacore T200 system (Cytiva, USA). Purified recombinant mouse Tnfaip2 protein (CSB-EP736815MO; CUSABIO, China) was immobilized on a Biacore CM5 sensor chip via standard amine coupling chemistry with an amine-coupling kit (Cytiva, USA). Serial dilutions of FA, ranging from 61.03 to 500,000 nM and prepared in 1 × PBS-P running buffer, were injected as the analyte over the chip surface. The resulting sensorgrams were processed and evaluated using the Biacore T200 Evaluation Software (version 2.0). The binding data were fitted to a 1:1 Langmuir binding model to determine the kinetic parameters.

### Statistical analyses

Statistical analyses were performed using GraphPad Prism 8. All data are presented as mean ± SME. The normality of data distribution was assessed using the Shapiro–Wilk test. For comparisons involving more than two groups, when data met the assumptions of normality and homogeneity of variances (tested by Levene’s test), one-way ANOVA followed by Tukey’s post hoc test was performed. If the data were not normally distributed or variances were unequal, the Kruskal–Wallis test followed by Dunn’s post hoc test was used. Results with *p* < 0.05 were considered significant.

## Supplementary Information

Supplementary material 1: Fig. 1. Knockdown of Tnfaip2 in BV2 cells alleviates TNFα-induced inflammation.mRNA expression levels of Tnfaip2, IL-1β, iNOS, and IL-6in BV2 cells transfected with Tnfaip2-siRNA for 24 h, followed by stimulation with TNFαfor 1 h.Tnfaip2 protein expression in BV2 cells treated as in.Ratio of phosphorylated p65 to total p65 protein in BV2 cells treated as in. Data are presented as mean ± SEM. *p < 0.05, **p < 0.01, ***p < 0.001; ns, not significant; n = 3 per group.

Supplementary material 2: Fig. 2. Gating strategy for Th1, Th2, and Th17 cells in flow cytometry. After excluding debris and doublets, CD3+ T lymphocytes were gated, from which CD4+ T cells were subsequently identified. Th1 cells were defined as CD4+IFN-γ+; Th2 cells as CD4+IL-4+; and Th17 cells as CD4+IL-17A+. The positivity threshold for each cytokine was set using fluorescence minus one controls.

## Data Availability

The datasets used and/or analysed during the current study are available from the corresponding author upon reasonable request.

## Abbreviations

BCA: Bicinchoninic acid assay
BFA: Brefeldin A
BSA: Bovine serum albumin
BTK: Bruton tyrosine kinase
CCK‑8: Cell Counting Kit‑8
cDNA: Complementary DNA
CFA: Complete Freund’s adjuvant
CNS: Central nervous system
COX‑2: Cyclooxygenase‑2
DAPI: 4′,6‑Diamidino‑2‑phenylindole
DMEM: Dulbecco's modified Eagle's medium
EAE: Experimental autoimmune encephalomyelitis
FA: Forsythoside A
FBS: Fetal bovine serum
FDR: False discovery rate
FITC: Fluorescein isothiocyanate
FPKM: Fragments per kilobase of transcript per million mapped reads
H&E: Hematoxylin and eosin
HRP: Horseradish peroxidase
Iba1: Ionized calcium‑binding adapter molecule 1
IFN‑γ: Interferon‑gamma
IL‑1β: Interleukin‑1 beta
IL‑6: Interleukin‑6
iNOS: Inducible nitric oxide synthase
K_D_: Equilibrium dissociation constant
KEGG: Kyoto Encyclopedia of Genes and Genomes
LFB: Luxol fast blue
LPS: Lipopolysaccharide
MBP: Myelin basic protein
MD: Molecular dynamics
MOG: Myelin oligodendrocyte glycoprotein
MS: Multiple sclerosis
NF‑κB: Nuclear factor kappa‑B
OCT: Optimal cutting temperature compound
PBS: Phosphate‑buffered saline
PE: Phycoerythrin
PMA: Phorbol 12‑myristate 13‑acetate
PMSF: Phenylmethylsulfonyl fluoride
PVDF: Polyvinylidene fluoride
qRT‑PCR: Quantitative real‑time polymerase chain reaction
R_max_: Maximum binding capacity
Rg: Radius of gyration
RMSD: Root mean square deviation
RMSF: Root mean square fluctuation
ROS: Reactive oxygen species
SASA: Solvent‑accessible surface area
SDS‑PAGE: Sodium dodecyl sulfate‑polyacrylamide gel electrophoresis
siRNA: Small interfering RNA
SPR: Surface plasmon resonance
TBST: Tris‑buffered saline with Tween‑20
Th1: T helper 1 cell
Th17: T helper 17 cell
Th2: T helper 2 cell
TNF‑α: Tumor necrosis factor alpha
Tnfaip2: TNF‑α‑induced protein 2
