# Supplementary figures and images for: Forsythoside A alleviates experimental autoimmune encephalomyelitis by targeting Tnfaip2
Source: Chin Med. 2026 Jul 29;21:204. doi: 10.1186/s13020-026-01476-z (PMC13418143; doi:10.1186/s13020-026-01476-z)

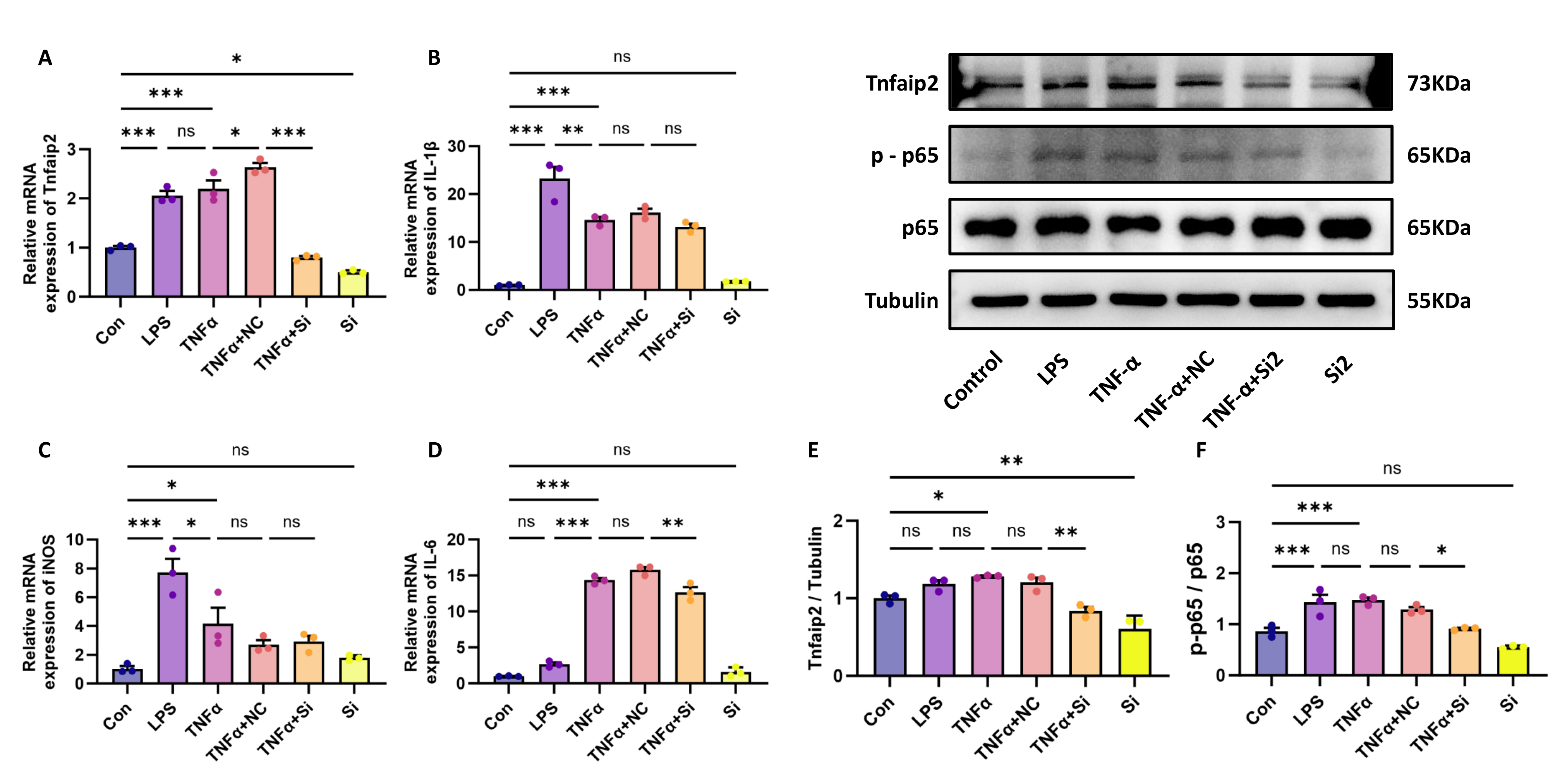

Supplement: Supplementary file 1 — Supplementary material 1: Fig. 1. Knockdown of Tnfaip2 in BV2 cells alleviates TNFα-induced inflammation.mRNA expression levels of Tnfaip2, IL-1β, iNOS, and IL-6in BV2 cells transfected with Tnfaip2-siRNA for 24 h, followed by stimulation with TNFαfor 1 h.Tnfaip2 protein expression in BV2 cells treated as in.Ratio of phosphorylated p65 to total p65 protein in BV2 cells treated as in. Data are presented as mean ± SEM. *p < 0.05, **p < 0.01, ***p < 0.001; ns, not significant; n = 3 per group. [file 13020_2026_1476_MOESM1_ESM.tif]

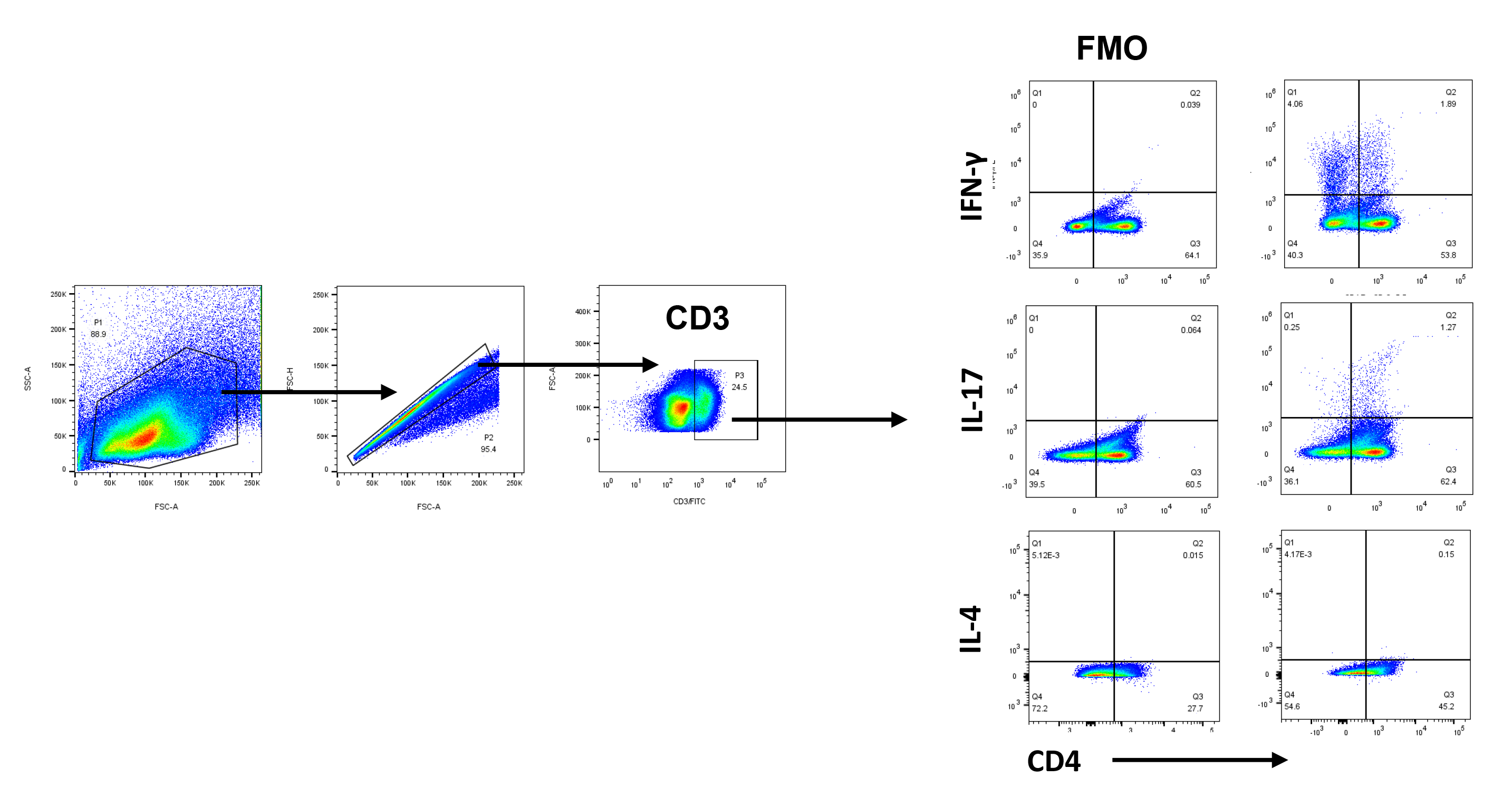

Supplement: Supplementary file 2 — Supplementary material 2: Fig. 2. Gating strategy for Th1, Th2, and Th17 cells in flow cytometry. After excluding debris and doublets, CD3+ T lymphocytes were gated, from which CD4+ T cells were subsequently identified. Th1 cells were defined as CD4+IFN-γ+; Th2 cells as CD4+IL-4+; and Th17 cells as CD4+IL-17A+. The positivity threshold for each cytokine was set using fluorescence minus one controls. [file 13020_2026_1476_MOESM2_ESM.tif]
